# Is Dry Needling Effective When Combined with Other Therapies for Myofascial Trigger Points Associated with Neck Pain Symptoms? A Systematic Review and Meta-Analysis

**DOI:** 10.1155/2021/8836427

**Published:** 2021-02-02

**Authors:** César Fernández-De-Las-Peñas, Gustavo Plaza-Manzano, Jorge Sanchez-Infante, Guido F Gómez-Chiguano, Joshua A Cleland, José L. Arias-Buría, Ibai López-de-Uralde-Villanueva, Marcos J. Navarro-Santana

**Affiliations:** ^1^Department of Physical Therapy, Occupational Therapy, Physical Medicine and Rehabilitation, Universidad Rey Juan Carlos (URJC), Alcorcón, Madrid, Spain; ^2^Cátedra Institucional en Docencia, Clínica e Investigación en Fisioterapia: Terapia Manual, Punción Seca y Ejercicio Terapéutico, Universidad Rey Juan Carlos, Alcorcón, Madrid, Spain; ^3^Department of Radiology, Rehabilitation and Physiotherapy, Universidad Complutense de Madrid, Madrid, Spain; ^4^Instituto de Investigación Sanitaria del Hospital Clínico San Carlos, Madrid, Spain; ^5^Performance and sport rehabilitation Laboratory, Faculty of sport sciences, University of Castilla-La Mancha, Toledo, Spain; ^6^Clínica Dinamia Fisioterapia, Madrid, Spain; ^7^Department of Public Health and Community Medicine, Tufts University School of Medicine, Boston, MA, USA; ^8^Rehabilitación San Fernando, Madrid, Spain

## Abstract

**Objective:**

To evaluate the effects of combining dry needling with other physical therapy interventions versus the application of the other interventions or dry needling alone applied over trigger points (TrPs) associated to neck pain. *Databases and Data Treatment*. Electronic databases were searched for randomized controlled trials where at least one group received dry needling combined with other interventions for TrPs associated with neck pain. Outcomes included pain intensity, pain-related disability, pressure pain thresholds, and cervical range of motion. The risk of bias (RoB) was assessed using the Cochrane risk of bias tool, methodological quality was assessed with PEDro score, and the quality of evidence was assessed by using the GRADE approach. Between-groups mean differences (MD) and standardized mean difference (SMD) were calculated.

**Results:**

Eight trials were included. Dry needling combined with other interventions reduced pain intensity at short-term (SMD −1.46, 95% CI −2.25 to −0.67) and midterm (SMD −0.38, 95% CI −0.74 to −0.03) but not immediately after or at long-term compared with the other interventions alone. A small effect on pain-related disability was observed at short-term (SMD −0.45, 95% CI −0.87 to −0.03) but not at midterm or long-term. The inclusion of dry needling was also effective for improving pressure pain thresholds only at short-term (MD 112.02 kPa, 95% CI 27.99 to 196.06). No significant effects on cervical range of motion or pain catastrophism were observed.

**Conclusion:**

Low-to-moderate evidence suggests a positive effect to the combination of dry needling with other interventions for improving pain intensity, pain-related disability, pressure pain thresholds, and cervical range of motion in people with neck pain associated with TrPs at short-term. No midterm or long-term effects were observed.

## 1. Introduction

Neck pain is the fourth ranked condition in number of years lived with disability [1] and has a lifetime prevalence of 70% and a point prevalence of 20% in the general population [2]. Physical therapy is often considered the first treatment option for people with neck pain. Different therapeutic strategies, e.g., cervical spine mobilizations and manipulations [3], thoracic manipulations [4], therapeutic exercise [5], or education [6], have shown to be effective for the treatment of neck pain. However, evidence supporting the use of other therapies proposed for the management of neck pain, such as dry needling, is still limited [7].

It is important to note that clinicians do not usually treat patients with neck pain with just one isolated intervention, and multimodal approaches are generally advocated. In fact, clinical practice guidelines for physical therapy management of people with neck pain recommend a combination of manual therapy combined with exercise as a potential therapeutic strategy for this population [8, 9]. Some systematic reviews have shown that the combination of two interventions seems to be more effective than the application of each intervention alone [10, 11]; however, others did not [12]. There are few systematic reviews and meta-analyses supporting an effect of dry needling for the management of neck pain [7, 13]. These reviews included trials investigating the isolated application of dry needling for patients with neck pain. No meta-analysis investigating the effects of adding dry needling to other physical therapy interventions for the management of trigger points (TrPs) associated to neck pain exists.

Therefore, the current systematic review and meta-analysis compares the effects of combining dry needling with other physical therapy interventions vs. application of other physical therapy interventions or dry needling alone applied over TrPs associated with neck pain symptoms.

## 2. Methods

This systematic review and meta-analysis adheres to the Preferred Reporting Items for Systematic Reviews and Meta-Analyses (PRISMA) statement [14]. The international OPS Registry registration link is https://doi.org/10.17605/OSF.IO/4J8H5.

### 2.1. Systematic Literature Search

Electronic literature searches were conducted on MEDLINE, CINAHL, PubMed, PEDro, Cochrane Library, SCOPUS, and Web of Science databases from their inception to 20 July 2020. When databases allowed limits, searches were restricted to randomized clinical trials. We also screened the reference lists of the identified trials. Bibliographical database search strategies were conducted with the assistance of an experienced health science librarian.

#### 2.1.1. Population

Adults with myofascial TrPs in the cervical muscles associated with neck pain symptoms of musculoskeletal origin older than 18 years of age.

#### 2.1.2. Intervention

Any form of muscular dry needling combined with other physical therapy interventions. Acupuncture was excluded.

#### 2.1.3. Comparators

Acceptable comparator was the other physical therapy intervention applied alone, the intervention combined with sham dry needling, or the application of just dry needling alone.

#### 2.1.4. Outcomes

The primary outcome measure was pain intensity or pain-related disability. Secondary outcomes included pressure pain thresholds or cervical range of motion. The search strategy for each database is available in Supplementary Table 1.

### 2.2. Selection Criteria

The systematic review included randomized clinical trials where at least one group received any form of dry needling combined with another intervention in people with TrPs associated with neck pain. Due to the heterogeneity in the terminology, we included the following diagnostic terms in the current meta-analysis: neck pain, myofascial neck pain, myofascial pain syndrome, and whiplash-associated pain.

The eligible criteria included adult population (>18 years old) with at least at one active TrP associated with neck pain symptoms, one group receiving dry needling targeting TrPs combined with other physiotherapy interventions, an acceptable comparator with other interventions alone or combined with sham/placebo or dry needling alone, and the primary outcome of the trial should include pain intensity (e.g., as measured with a visual analogue scale or numerical pain rate scale) or pain-related disability (e.g., as assessed with a specific-disease questionnaire). Secondary outcomes included pain sensitivity (e.g., pressure pain thresholds) or cervical range of motion (e.g., assessed with a goniometer). We excluded clinical trials including pain associated with neurological disorders (e.g., poststroke pain), postoperative neck pain and studies not published as a journal article, retrospective designs, pilot studies, needling using a traditional Chinese medicine approach, or use of injection therapy (e.g., lidocaine injection).

### 2.3. Screening, Selection Process, and Data Extraction

Articles identified from the different databases were independently reviewed by two authors. First, the duplicates were removed. Second, title and abstract of the articles were screened for potential eligibility. Third, a full-text read of potentially eligible studies was conducted. Authors were required to achieve a consensus on the included trials. In case of discrepancy between both reviewers, a third author participated in the process to reach the consensus for including or not including the study.

Data from each trial including study design, sample size, population, interventions, outcomes, and follow-ups were extracted independently by 2 authors in a standardized form. Both authors had to achieve a consensus on each item on the data-extraction form. If disagreement occurred, a third author participated in the determination.

### 2.4. Assessment of Methodological Quality and Risk of Bias

Risk of bias and methodological quality of the included trials were independently assessed by two authors using the Cochrane risk of bias (RoB) assessment tool [15] and the Physiotherapy Evidence Database (PEDro) scale [16], respectively.

The RoB tool includes the following items: selection bias (randomization sequence generation and allocation concealment), performance bias (blinding participants and blinding therapists), detection bias (blinding outcome assessor), attrition bias (incomplete outcome data), reporting bias (source of funding bias/selecting outcome reporting), and other bias (sample size) [15]. Each item was classified as low risk, high risk, or unclear according to the Cochrane collaboration's tool [15].

The PEDro score evaluates the quality of the trial by assessing the following items: random allocation, concealed allocation, baseline between-groups similarity, participants blinding, therapists blinding, assessors blinding, dropouts, intention-to-treat statistical analysis, between-groups statistical comparison, point measures, and variability data [16]. A trial was considered of high-quality when the PEDro score was ≥6 over 10 points.

### 2.5. Level of Evidence

To evaluate the quality of the evidence, we used the Grading of Recommendations Assessment, Development, and Evaluation (GRADE) approach [17]. The evidence level was classified as high, moderate, low, or very low based on the following items: presence of the study limitations (RoB), indirectness of evidence, inconsistency of results/unexplained heterogeneity, imprecision of results, and high probability of publication bias [18]. The level of evidence was classified as high quality when all items were negative, moderate quality when one item included serious risk, low quality when two items showed serious risk or one item showed very serious risk, or very low quality when three or more items have serious risk or two or more showed very serious risk. This process was also independently performed by two authors, with the participation of a third one if discrepancy occurred.

### 2.6. Data Synthesis and Analysis

The meta-analysis was conducted using the Review Manager statistical software (RevMan version 5.3). Data synthesis was presented by groups according to the inclusion of TrP dry needling with other interventions vs. the same intervention alone or vs. TrP dry needling alone and by the follow-up period as immediately after, at short-term, midterm, and long-term, if data were available.

We extracted the sample size, means, and standard deviations for each variable. When the trial reported only standard errors, they were converted to standard deviations. When necessary, the mean scores and standard deviations were estimated from graphs. Also, if the trial presented nonparametric values (median and interquartile range), they were converted to means and standard deviations [19, 20].

The between-groups mean differences (MD) of the trials were converted to SMD, with their 95% confidence intervals (CI). A random-effects model was used to determine the overall effect size (SMD). An effect size (SMD) of 0.8 or greater was considered large, between 0.5 and 0.8 as moderate, and between 0.2 and 0.5 as small. In general, *P* values < 0.05 were considered statistically significant [21]. The calculation of the effect size on pain and related-disability were obtained immediate after (less than one week) just one session and at short-term (1–12 weeks), midterm (12–24 weeks), and long-term (>24 weeks).

Cervical range of motion was pooled for each movement, i.e., flexion, extension, lateral-flexion, and rotation. When the trial calculated the total range of motion or either side separately for lateral-flexion and rotation, the mean was used in the main analysis.

The heterogeneity of the studies was assessed using the *I*^2^ statistic. The Cochrane group has established the following interpretation of the *I*^2^ statistic: 0%–40% may not be relevant/important heterogeneity; 30%–60% suggests moderate heterogeneity, 50%–90% represents substantial heterogeneity, and 75–100% considerable heterogeneity [22].

## 3. Results

### 3.1. Study Selection

**Figure 1:**
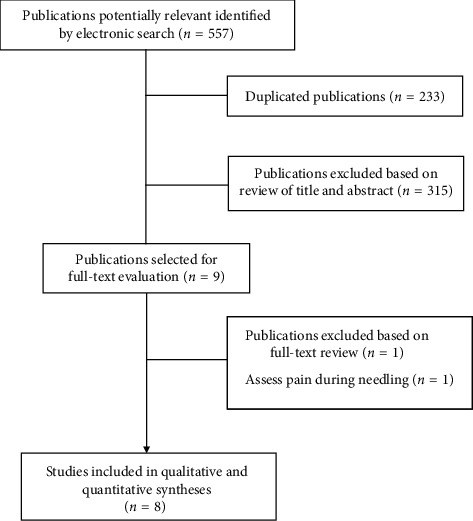
Preferred Reporting Items for Systematic Reviews and Meta-Analyses (PRISMA) Flow diagram.

The electronic searches identified 557 potential studies for review. After removing duplicates, 324 studies remained. Three hundred fifteen (*n* = 315) were excluded based on examination of their titles or abstracts, leaving 9 articles for full-text analysis [23–31]. One trial was excluded due to the objective of the study was to observe the effectiveness on postneedling soreness [23]. A total of 8 trials [24–31] were included in the systematic review and in the quantitative analysis (Figure 1).

### 3.2. Study Characteristics

The characteristics of the participants of the included studies are shown in Table 1. All studies targeted active TrPs (i.e., those which referred pain reproduced the patient's symptoms) with the needle, five (62.5%) targeted TrPs in the posterior neck muscles from a pragmatic viewpoint [25–27, 29, 31], two just the upper trapezius muscle [24, 30], and the last one the upper trapezius and levator scapulae [28]. Although all trials included one group receiving dry needling, two did not report the presence of local twitch responses during the needling intervention [26, 27]. All clinical trials specified that dry needling was applied by a physical therapist. The combination of the interventions was grouped since six trials compared the combination of dry needling with other interventions against the application of that intervention alone [26–31], and the remaining two compared the combination of dry needling with other interventions against dry needling alone [24, 25]. There was heterogeneity in the complementary interventions since three trials used best evidence-based physical therapy approaches [26, 28, 31], two trials included just stretching [29, 30], one just exercise [27], one pain neuroscience education [25], and the last one the application of percutaneous electrical nerve stimulation [24] (Table 1). All trials included pain intensity as the primary outcome, whereas six (62.5%) also assessed pain-related disability. Secondary outcomes (pressure pain thresholds and cervical range of motion) were assessed in five trials. In addition, pain catastrophizing was also assessed in three trials [25, 27, 31]; therefore, pooling data were also conducted. Supplementary Table 2 summarizes the characteristics of dry needling interventions applied in each trial.

## 4. Methodological Quality

The methodological quality scores ranged from 6 to 9 (mean: 7.2; SD: 1.1) out of a maximum of 10 points; therefore, all studies were considered of high methodological quality (≥6 points). No trial was able to blind the therapists. The most frequent bias was blinding participants since only three trials were able to do [26–28].  Table 2 represents the details of the PEDro scale of each trial.

### 4.1. Risk of Bias

**Figure 2:**
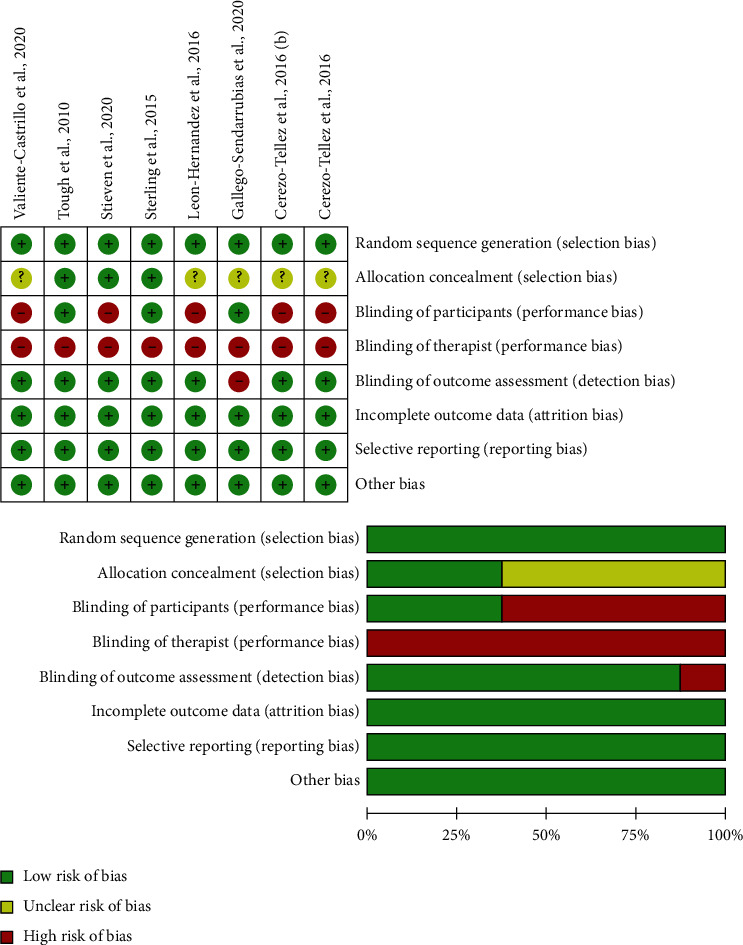
Plot of risk of bias of the included studies.

The details of the risk of bias assessment of the included trials are displayed in Figure 2. No trial was able to blind therapists, and all trials had an unclear bias in the item of blinding participants. In general, the risk of bias of the included trials in the current meta-analysis was low.

### 4.2. Dry Needling Combined with Other Therapies on Pain Intensity

**Figure 3:**
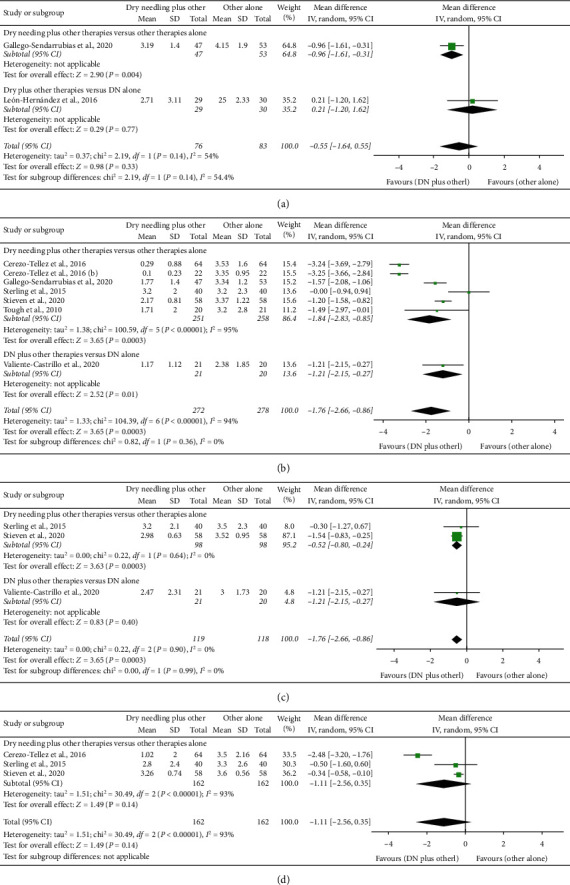
Comparison (mean differences) between the effects of dry needling combined with other interventions against other interventions on pain intensity (a) immediately after, (b) at short-term, (c) at midterm, and (d) at long-term.

Dry needling combined with other physical therapy interventions did not exhibit a significant effect (MD −0.55 points, 95% CI −1.64 to 0.55, *P*=0.33, *Z* = 0.98, *n* = 159) for reducing pain intensity immediately after one single treatment session when compared with other interventions or dry needling alone, although this analysis was based on just one trial each (Figure 3(a)).

At short-term follow-up, the meta-analysis found that dry needling combined with other interventions showed a significant large effect (MD −1.76 points, 95% CI −2.66 to −0.86; SMD −1.46, 95% CI −2.25 to −0.67, *P*=0.001, *Z* = 3.83, *N* = 550, 6 trials) for reducing pain intensity as compared to the other interventions alone or dry needling alone but with considerable heterogeneity (*I*^2^ = 94%) between the studies (Figure 3(b)). The effect was positive in both comparisons, dry needling combined with other interventions vs. the other interventions alone (MD −1.84 points, 95% CI −2.83 to −0.85), and dry needling with other interventions vs. dry needling alone (MD −1.21 points, 95% CI −2.15 to −0.27).

The results revealed that dry needling combined with other interventions exhibited a significant small effect (MD −0.52 points, 95% CI −0.79 to −0.25; SMD −0.38, 95% CI −0.74 to −0.03, *P*=0.002, *Z* = 3.72, *n* = 237) for decreasing pain intensity at midterm than the other interventions or dry needling alone and without heterogeneity (*I*^2^ = 0%) between the trials (Figure 3(c)). The effect was significant for dry needling combined with other interventions vs. the other interventions alone (MD −0.52 points, 95% CI −0.80 to −0.24) but not for dry needling combined with other therapy vs. dry needling alone (MD −0.53 points, 95% CI −1.78 to 0.25).

No significant effect on pain (MD −1.30 points, 95% CI −3.27 to 0.66; SMD −0.64, 95% CI −1.20 to −0.08, *P*=0.19, *Z* = 1.30, *n* = 324) was observed at the long-term follow-up for the inclusion of dry needling with other interventions (Figure 3(d)). Furthermore, considerable heterogeneity between the trials was observed (*I*^2^ = 98%).  Table 3 summarizes the main results and raw data of the included studies.

### 4.3. Dry Needling Combined with Other Therapies on Related-Disability

**Figure 4:**
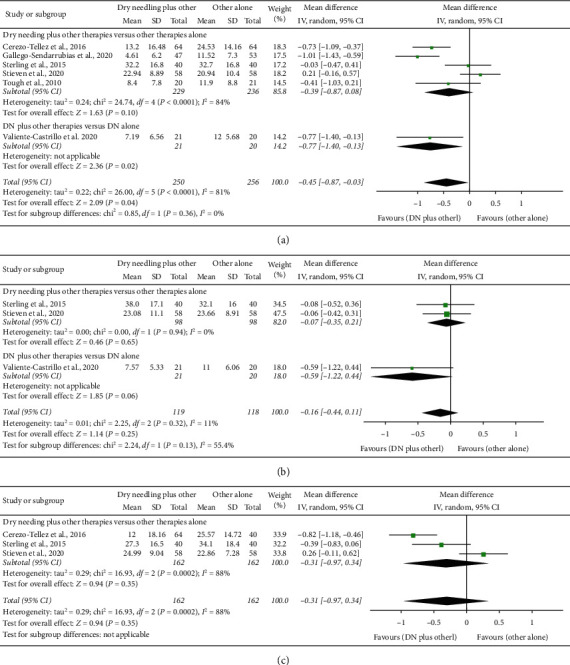
Comparison (standardized mean differences) between the effects of dry needling combined with other interventions against other interventions on pain-related disability (a) at short-term, (b) at midterm, and (c) at long-term.

A significant effect on related-disability for the combination of dry needling with other interventions was observed at short-term (SMD −0.45, 95% CI −0.87 to −0.03, *P*=0.5, *Z* = 2.09, *n* = 506, Figure 4(a)) but not at midterm (SMD −0.16, 95% CI −0.44 to 0.11, *P*=0.25, *Z* = 1.14, *n* = 237, Figure 4(b)) and long-term (SMD −0.32, 95% CI −0.97 to 0.29, *P*=0.35, *Z* = 0.94, *n* = 324, Figure 4(c)). The heterogeneity between trials was considerable (*I*^2^ = 81%) at short-term, not relevant (*I*^2^ = 11%) at midterm, and considerable (*I*^2^ = 88%) at long-term.

At short-term, a significant effect on pain-related disability was found when compared the combined application of dry needling against dry needling alone (SMD −0.77, 95% CI −1.40 to −0.13), but this analysis was based on just one trial (Figure 4(a)).  Table 3 details the main results and raw data of the included studies.

### 4.4. Dry Needling Combined with Other Therapies on Pressure Pain Thresholds

**Figure 5:**
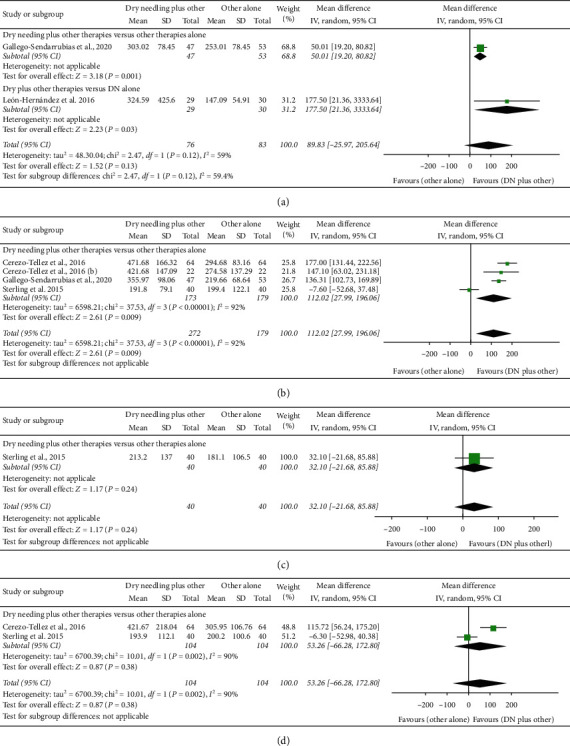
Comparison (mean differences) between the effects of dry needling combined with other interventions against other interventions on pressure pain thresholds (a) immediately after, (b) at short-term, (c) at midterm, and (d) at long-term.

The meta-analysis found that dry needling in combination with other therapies did not exhibit a significant effect for increasing pressure pain thresholds immediately after (MD 89.93 kPa, 95% CI −25.97 to 205.64, *P*=0.13, *Z* = 1.52, *n* = 159, Figure 5(a)), at midterm (MD 32.10 kPa, 95% CI −21.68 to 85.88, *P*=0.24, *Z* = 1.17, *n* = 80, Figure 5(c)), and at long-term (MD 53.26 kPa, 95% CI −66.28 to 172.80, *P*=0.38, *Z* = 0.87, *n* = 208, Figure 5(d)).

At short-term, dry needling combined with other therapies exhibited a significant effect (MD 112.02 kPa, 95% CI 27.99 to 196.06, *P*=0.009, *Z* = 2.61, *n* = 352) for increasing pressure pain threshold when compared with the other interventions alone, although with considerable heterogeneity (I^2^ = 92%) between the studies (Figure 5(b)).

### 4.5. Dry Needling Combined with Other Therapies on Cervical Range of Motion

**Figure 6:**
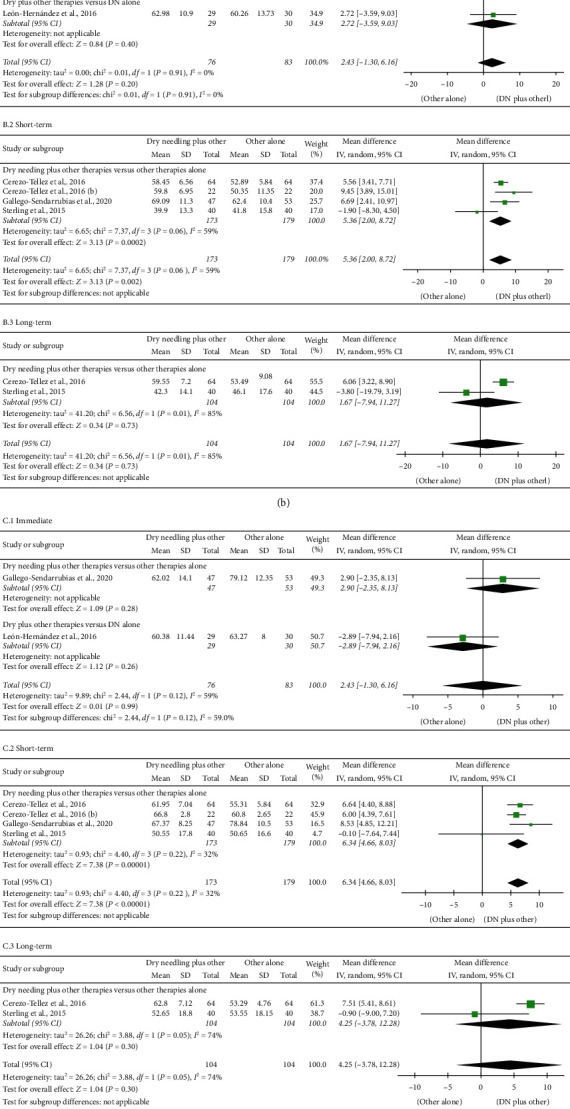
Comparison (mean differences) between the effects of dry needling combined with other interventions against other interventions on cervical range of motion in flexion (a), extension (b), rotation (c), and lateral-flexion (d) motion (1) immediately after, (2) at short-term, and (3) at long-term.

Dry needling combined with other interventions did not show a significant effect immediately after the intervention on the cervical range of motion when compared with the other interventions alone: flexion (MD 3.33, 95% CI −0.28 to 6.97, *n* = 159, *Z* = 1.81, *P*=0.08, Figure 6(a). 1); extension (MD 2.43, 95% CI −1.30 to 6.16, *n* = 159, *Z* = 1.28, *P*=0.20, Figure 6(b). 1); rotation (MD −0.03, 95% CI −5.71 to 5.64, *n* = 159, *Z* = 0.01, *P*=0.99, Figure 6(c). 1); and lateral-flexion (MD 2.13, 95% CI −1.14 to 5.41, *n* = 159, *Z* = 1.28, *P*=0.20, Figure 6(d)). Similarly, no significant effects at long-term were either observed for flexion (MD 2.89, 95% CI −4.67 to 10.45, *n* = 208, *Z* = 0.75, *P*=0.45, Figure 6(a). 3); extension (MD 1.67, 95% CI −7.94 to 11.27, *n* = 208, *Z* = 0.34, *P*=0.73, Figure 6(b). 3); and rotation (MD 4.25, 95% CI −3.78 to 12.26, *n* = 208, *Z* = 1.04, *P*=0.30, Figure 6(c). 3) for the combination of dry needling and other interventions. A significant effect at long-term was seen for lateral-flexion (MD 5.89, 95% CI 3.72 to 8.06, *n* = 128, *Z* = 5.32, *P* < 0.001, Figure 6(c). 3), although this analysis was based on just one study.

The meta-analysis observed a significant small short-term effect of dry needling combined with other interventions on the cervical range of motion: flexion (MD 6.01, 95% CI 2.86 to 9.16, *n* = 352, *Z* = 3.74, *P* < 0.001, Figure 6(a). 2); extension (MD 5.36, 95% CI 2.00 to 8.72, *n* = 352, *Z* = 3.13, *P*=0.002, Figure 6(b). 2); rotation (MD 6.34, 95% CI 4.661 to 8.03, *n* = 352; *Z* = 7.38, *P* < 0.001, Figure 6(c). 2); lateral-flexion (MD 8.55, 95% CI 5.01 to 12.10, *n* = 272, *Z* = 4.73, *P* < 0.001, Figure 6(d). 2). All analyses had moderate heterogeneity.  Table 3 summarizes main results and raw data of the included studies.

### 4.6. Dry Needling Combined with Other Therapies on Pain Catastrophizing

**Figure 7:**
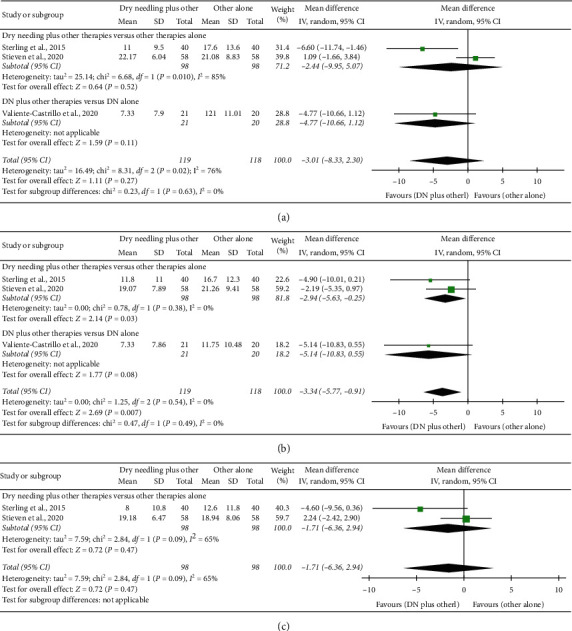
Comparison (mean differences) between the effects of dry needling combined with other interventions against other interventions on pain catastrophism (a) at short-term, (b) at midterm, and (c) at long-term.

The combination of dry needling with other therapies exhibits a significant small effect on pain catastrophism at midterm (MD −1.71, 95% CI −6.36 to 2.94; SMD -0.36, 95% CI −0.61 to −0.10, *n* = 237; *Z* = 2.69; *P*=0.007, Figure 7(b)) but not at short-term (MD −3.01, 95% CI −8.33 to 2.30, *n* = 237; *Z* = 1.11; *P*=0.27, Figure 7(a)) and long-term (MD −3.34, 95% CI −5.77 to −0.91; *n* = 196; *Z* = 0.72; *P*=0.47, Figure 7(c)).

### 4.7. Quality of Evidence (GRADE)

Table 4 displays the details of GRADE assessment showing RoB, inconsistency of the results, indirectness of evidence, imprecision of results, and high probability of publication bias. The serious/very serious inconsistency of the results (heterogeneity) and the serious/very serious impression downgraded the evidence level of dry needling to low or very low.

### 4.8. Adverse Events

Seven trials (87.5%) reported information about adverse effects with all of them reporting just minor events and none reported any serious adverse effects. Postneedling soreness was the most common adverse event in all trials and resolved spontaneously in 24–48 h without further treatment (Supplementary Table 3).

## 5. Discussion

### 5.1. Trigger Point Dry Needling Combined with Other Therapies

The objective of this meta-analysis was to compare the effects of the application of dry needling combined with other interventions against an intervention alone or dry needling alone applied over cervical TrPs associated with neck pain symptoms. We found low-to-moderate evidence suggesting a positive effect of including dry needling into physical therapy treatment for improving pain intensity at short-term and midterm and for improving pain-related disability at short-term as compared with the physical therapy intervention alone. Additionally, adding dry needling to a physical therapy intervention was also effective at short-term but not midterm and long-term, for increasing pressure pain thresholds and cervical range of motion. A small effect on pain catastrophism at midterm was found. The RoB of the clinical trials included in this study was generally low, but the inconsistency (heterogeneity) and imprecision of the results downgraded the level of evidence (GRADE).

The current meta-analysis is the first one investigating the impact of dry needling combined other interventions versus another intervention alone on pain intensity, related-disability, pressure pain sensitivity, cervical range of motion, and pain catastrophism in patients with TrPs associated with neck pain symptoms. Liu et al. [7] investigated the effects of the isolated application of dry needling and found low evidence supporting its effects immediately after and at 4 weeks when compared with control or sham. We found low-quality evidence supporting a small positive effect of the inclusion of dry needling into a physical therapy treatment for improving pain intensity and pain-related disability when compared with the physical therapy treatment approach alone; however, the effects were observed mostly at short-term and at midterm only for pain intensity. The decrease on pain of −0.96 points (95% CI −1.61 to −0.31) at short-term and of −1.84 points (95% CI −2.83 to −0.85) at midterm did not reach the minimal clinically important difference (MCID) of 2.1 points described for people with mechanical neck pain [32], although changes at midterm were slightly superior to the general MCID of 1.4 points determined by Bijur et al. [33]. Nevertheless, we should recognize that the lower bound estimate of the confidence intervals did not surpass the MCID in either case, limiting the clinical relevance of these results. It is possible that some patients with TrPs associated with neck pain symptoms exhibit more benefits to dry needling than others. Based on current evidence, it seems that including dry needling into a physical therapy treatment approach could have only small effects at short-term and midterm follow-up periods for the treatment of neck pain associated to TrPs (low-to-moderate evidence); however, more studies are clearly needed.

We also found that adding dry needling into a physical therapy intervention has a moderate effect (low evidence) at short-term for decreasing pressure pain sensitivity (by increasing the pressure pain thresholds) and small effects for increasing cervical range of motion. These results agree with current theories supporting a potential hypoalgesic effect of dry needling [34], although differences were only significant for short-term. It is possible that this neurophysiological effect is short-lasting. On the contrary, the effects of adding dry needling on cervical range of motion were small and should not be considered as clinically relevant. These results may be related to the fact that most trials included in the current meta-analysis have shown positive effects on these outcomes, and the inclusion of another intervention does not lead to better results, which has been also found when combining manual therapy with exercise for the management of neck pain [12]. This can be also related to the fact that manual therapy approaches [35] and dry needling interventions [34] share common neurophysiological mechanisms, and they only potentiate their effects on a subgroup of patients. Future studies should investigate this.

### 5.2. Safety of Trigger Point Needling

Since dry needling is an invasive intervention, clinicians should monitor the presence of adverse events. Carlesso et al. [36] defined an adverse event “as a sequela of medium-term duration with any symptom perceived as unacceptable to the patient and requiring further treatment.” Adverse events can be categorized as minor, moderate, or major. Previous studies have found that most events occurring after application of dry needling, such as bleeding or postneedling soreness, can be categorized as minor adverse events [37, 38]. Most studies included in this meta-analysis monitored the presence of adverse events during the study and reported the presence of postneedling soreness as the most common adverse event, supporting that dry needling seems to be a potentially safe intervention. Nevertheless, major adverse events, e.g., pneumothorax, have been also reported in the literature when applied dry needling to the cervical and thoracic spine, although their rate is less than 0.1% (1 per 1,024 needling treatments) and depend on the anatomical location. In fact, case reports describing pneumothorax after dry needling treatment have applied the intervention over thoracic musculature [39, 40]. Although dry needling seems to be a safe intervention if properly applied, therapists need to be aware of the potential risks associated with its application on each body area where it is applied.

### 5.3. Strengths and Limitations

The results of the current meta-analysis should be generalized within the context of its potential strengths and limitations. The strengths include a comprehensive literature search, methodological rigor, exhaustive data extraction, rigorous statistical analysis, and the inclusion of randomized controlled trials of high methodological quality. Among the limitations, we recognized that dry needling was applied with different dosages, that is, sessions, frequency of application, and combined with a variety of interventions exhibiting different evidence (e.g., manual therapy, stretching, and exercise). Second, the heterogeneity and imprecision of the results of the trials was serious; therefore, current results should be taken with caution. Third, the number of trials in some comparisons was small (*n* = 3) which limits the extrapolation of the results. It is possible that a greater number of high-quality clinical trials investigating midterm and long-term effects of dry needling combined with more detailed physical therapy interventions would lead to different results.

### 5.4. Clinical and Research Implications

Although this is the first meta-analysis investigating the effects of adding dry needling to other physical therapy interventions in patients with neck pain associated to myofascial TrPs, several questions remain to be elucidated. First, just few studies investigating long-term follow-up periods are available in the literature. Second, trials in this meta-analysis investigated different physiotherapy approaches in heterogeneous populations (traumatic vs. insidious onset). Third, since neck pain is characterized by motor control disturbances, the inclusion of dry needling could lead to changes in muscle strength outcomes in this population. A recent meta-analysis reported medium effect sizes for dry needling to enhance force production in individuals with neck pain (moderate evidence), although this analysis was just based on two studies [41]. In fact, these two studies were included in the current meta-analysis, but we did not pool data from strength outcomes due to the heterogeneous interventions applied in them. It is probable that the combination of dry needling would be not as effective as it can be with any physical therapy intervention. Proper understanding of the clinical presentation of each individual patient and the underlying mechanisms of each intervention could lead to better clinical outcomes.

## 6. Conclusion

The current meta-analysis found low-to-moderate evidence suggesting a positive effect of adding dry needling into a physical therapy approach for improving pain intensity at short-term and midterm and for improving pain-related disability at short-term as compared with the same intervention applied alone. Additionally, adding dry needling was effective at short-term for increasing pressure pain thresholds and cervical range of motion and on pain catastrophism at midterm. Although the methodological quality of the included trials was high, the inconsistency (heterogeneity) and imprecision of the results downgraded the overall levels of evidence.

## Figures and Tables

**Table 1:** Characteristics of the sample of included studies.

Study	Diagnosis	Group	Total (male/female)	Age (SD), y	Pain duration
*DN plus other therapies vs. other therapies alone*					
Tough et al., 2010 [26]	Whiplash-associated disorders	G1: TrP-DN + standardized physical therapy	20 (9/11)	34.2 (10.8)	6.8 (4.3) wk.
		G2: sham DN + standardized physical therapy	21 (8/13)	36.9 (10.9)	7.3 (4.7) wk.

Sterling et al., 2015 [27]	Chronic whiplash-associated disorders	G1: TrP-DN + exercise therapy	40 (16/24)	41.5 (11.1)	20.6 (18.0) mo.
		G2: sham TrP-DN + exercise therapy	40 (10/30)	41.7 (12.3)	15.9 (12.8) mo.

Cerezo Tellez et al., 2016 [29]	Chronic mechanical neck pain	G1: TrP-DN + passive stretching	64	48 (15.7)	>6 mo.
		G2: passive stretching	64	52 (16.6)	>6 mo.

Cerezo-Tellez et al., 2016 [30]	Neck pain in office workers	G1: TrP-DN + passive stretching	22 (5/17)	40.1 (13.1)	NR
		G2: passive stretching	22 (3/19)	47 (16.2)	NR

Gallego-Sendarrubias et al., 2020 [28]	Chronic mechanical neck pain	G1: TrP-DN + manual therapy	47 (13/34)	34.1 (7.6)	>3 mo.
		G2: sham TrP-DN + manual therapy	53 (24/29)	34.6 (8.9)	>3 mo.

Stieven et al., 2020 [31]	Chronic neck pain	G1: TrP-DN + guideline based physical therapy	58 (14/44)	39.3 (9.9)	36.1 (12.4) mo.
		G2: guideline based physical therapy	58 (18/40)	36.9 (11.5)	41.6 (14.1) mo.

*DN plus other therapies vs. DN alone*					
León-Hernández et al., 2016 [24]	Chronic myofascial neck pain	G1: DN alone	31 (7/24)	23.32 (4.77)	16.03 (17.23) mo.
		G2: DN + PENS	31 (9/22)	26.81 (9.63)	19.36 (19.23) mo.

Valiente-Castrillo et al., 2020 [25]	Chronic myofascial neck pain	G1: TrP-DN	20 (4/16)	40.33 (11.94)	43.39 (56.54) mo.
		G2: TrP-DN + pain neuroscience education	21 (2/19)	40.35 (7.97)	64.94 (62.93) mo.
		G3: usual care (N/A)	19 (3/16)	42.35 (9.43)	56.29 (67.74) mo.

TrP, trigger point; DN, dry needling; SDN, superficial dry needling; PENS, percutaneous electrical nerve stimulation; G, group; Y, years; NR, not reported; mo., months; wk., weeks.

**Table 2:** Score of randomized clinical trials with the PEDro scale.

	1	2	3	4	5	6	7	8	9	10	Total
Tough et al., 2010 [26]	Y	Y	Y	Y	N	Y	N	Y	Y	Y	8/10
Sterling et al., 2015 [27]	Y	Y	Y	Y	N	Y	Y	Y	Y	Y	9/10
Cerezo-Tellez et al., 2016 [29]	Y	N	Y	N	N	Y	Y	N	Y	Y	6/10
Cerezo-Tellez et al., 2016 [30]	Y	N	Y	N	N	Y	Y	N	Y	Y	6/10
León-Hernández et al., 2016 [24]	Y	N	Y	N	N	Y	Y	Y	Y	Y	7/10
Gallego-Sendarrubias et al., 2020 [28]	Y	N	Y	Y	N	N	Y	N	Y	Y	6/10
Stieven et al., 2020 [31]	Y	Y	Y	N	N	Y	Y	Y	Y	Y	8/10
Valiente-Castrillo et al., 2020 [25]	Y	N	Y	N	N	Y	Y	Y	Y	Y	7/10

1, random allocation of participants; 2, concealed allocation; 3, similarity between groups at baseline; 4, participant blinding; 5, therapist blinding; 6, assessor blinding; 7, fewer than 15% dropouts; 8, intention-to-treat analysis; 9, between-group statistical comparisons; 10, point measures and variability data.

**Table 3:** Main results and raw data of the included studies.

Study	Outcome/Group	Baseline mean (SD)	Immediate, less than one week after a single session	Short-term, 1–12 weeks, mean (SD)	Midterm, 12–24 weeks, mean (SD)		Long-term, >24 weeks, mean (SD)		
*Dry needling combined with other interventions vs. other intervention alone*									
Tough et al., 2010 [26]	Pain (VAS, 0–10)	4.9 (1.6)	—	1.71 (2.0) (6 wk)	—		—		
	G1	5.0 (1.6)	—	3.2 (2.8) (6 wk)	—		—		
	G2	18.6 (8.7)	—	8.4 (7.8) (6 wk)	—		—		
	Disability (NDI)								
	G1	20.5 (7.6)	—	11.9 (8.8) (6 wk)	—		—		
	G2		—		—		—		

Sterling et al., 2015 [27]	Pain (NPRS, (0–10)								
	G1	5.6 (2.2)	—	3.2 (2.0) (6 wk)	3.2 (2.1) (12 wk)		2.8 (2.4) (54 wk)		
	G2	5.4 (2.0)	—	3.2 (2.3) (6 wk)	3.5 (2.3) (12 wk)		3.3 (2.6) (54 wk)		
	Disability (NDI, %)								
	G1	42.9 (15.2)	—	32.2 (16.8) (6 wk)	30.8 (17.1) (12 wk)		27.3 (16.5) (54 wk)		
	G2	42.9 (13.1)	—	32.7 (16.8) (6 wk)	32.1 (16.0) (12 wk)		34.1 (18.4) (54 wk)		
	Cervical flexion (°)								
	G1	39.7 (16.1)	—	39.2 (15.8) (6 wk)	41.7 (18.7) (12 wk)		42.5 (16.5) (54 wk)		
	G2	39.7 (14.9)	—	41.2 (19.1) (6 wk)	39.6 (16.5) (12 wk)		44.3 (17.0) (54 wk)		
	Cervical extension (°)								
	G1	33.3 (13.2)	—	39.9 (13.3) (6 wk)	39.8 (13.6) (12 wk)		42.3 (14.1) (54 wk)		
	G2	36.3 (16.3)	—	41.8 (15.8) (6 wk)	40.8 (16.1) (12 wk)		46.1 (17.6) (54 wk)		

Sterling et al., 2015 [27]	Right cervical rotation (°)								
	G1	45.8 (17.6)	—	51.6 (18.4) (6 wk)	52.0 (19.5) (12 wk)		54.0 (19.2) (54 wk)		
	G2	42.0 (22.5)	—	52.5 (17.4) (6 wk)	48.8 (17.9) (12 wk)		55.7 (17.7) (54 wk)		
	Left cervical rotation (°)								
	G1	43.6 (16.3)	—	49.5 (17.2) (6 wk)	49.7 (20.3) (12 wk)		51.3 (18.4) (54 wk)		
	G2	46.3 (15.1)	—	48.8 (15.8) (6 wk)	46.4 (16.3) (12 wk)		51.4 (18.6) (54 wk)		
	Cervical rotation (mean calculated)								
	G1	44.7 (16.95)	—	50.55 (17.8) (6 wk)	50.85 (19.9) (12 wk)		52.65 (18.8) (54 wk)		
	G2	44.15 (18.8)	—	50.65 (16.6) (6 wk)	47.6 (17.1) (12 wk)		53.55 (18.15) (54 wk)		
	PPT (kPa)								
	G1	153.8 (100.4)	—	191.8 (79.1) (6 wk)	213.2 (137.0) (12 wk)		193.9 (112.1) (54 wk)		
	G2	174.7 (108.5)	—	199.4 (122.1) (6 wk)	181.1 (106.5) (12 wk)		200.2 (100.6) (54 wk)		
	Pain catastrophizing (PCS)								
	G1	18.0 (11.3)	—	11.0 (9.5) (6 wk)	11.8 (11.0) (12 wk)		8.0 (10.8) (54 wk)		
	G2	20.4 (13.8)	—	17.6 (13.6) (6 wk)	16.7 (12.3) (12 wk)		12.6 (11.8) (54 wk)		

Cerezo Tellez et al., 2016 [29]	Pain (VAS, 0–10)								
	G1	5.1 (1.6)	—	0.29 (0.88) (2 wk)	—		1.02 (2) (24 wk)		
	G2	5.1 (1.4)	—	3.53 (1.6) (2 wk)	—		3.5 (2.16) (24 wk)		
	PPT (kPa) right trapezius								
	G1	205.93 (68.64)	—	480.51 (172.64) (2 wk)	—		429.52 (212.08) (24 wk)		
	G2	196.13 (58.83)	—	292.23 (86.32) (2 wk)	—		304.98 (125.52) (24 wk)		
	PPT (kPa) left trapezius								
	G1	205.93 (78.45)	—	462.86 (160) (2 wk)	—		413.82 (224) (24 wk)		
	G2	205.93 (78.45)	—	297.13 (80) (2 wk)	—		306.93 (88) (24 wk)		
	PPT (kPa) mean calculated								
	G1	205.93 (73.54)	—	471.68 (166.32) (2 wk)	—		421.67 (218.04)		
	G2	201.03 (68.64)	—	294.68 (83.16) (2 wk)	—		305.95 (106.76)		

Cerezo Tellez et al., 2016 [29]	Cervical rotation (°)								
	G1	53.9 (11.25)	—	61.95 (7.04) (2 wk)	—		62.8 (7.12) (24 wk)		
	G2	53.3 (9.05)	—	55.31 (5.84) (2 wk)	—		55.29 (4.76) (24 wk)		
	Cervical lateral-flexion (°)								
	G1	31.55 (8.1)	—	38.09 (6.56) (2 wk)	—		38.3 (6.76) (24 wk)		
	G2	29.95 (6.75)	—	31.96 (4.16) (2 wk)	—		32.41 (5.72) (24 wk)		
	Cervical flexion-extension (°)								
	G1	49.9 (9.45)	—	58.45 (6.56) (2 wk)	—		59.55 (7.2) (24 wk)		
	G2	51 (8.3)	—	52.89 (5.84) (2 wk)	—		53.49 (9.08) (24 wk)		
	Disability (NDI)								
	G1	30.5 (16)	—	13.2 (16.48) (2 wk)	—		12 (18.16) (24 wk)		
	G2	31 (12)	—	24.53 (14.16) (2 wk)	—		22.57 (14.72) (24 wk)		

Cerezo-Tellez et al., 2016 [30]	Pain (VAS, 0–10)^+^								
	G1	5.8 (0.79)	—	0.10 (0.23) (2 wk)	—		—		
	G2	5.0 (1.34)	—	3.35 (0.95) (2 wk)	—		—		
	PPT (kPa)								
	G1	186.32 (68.64)	—	421.68 (147.09) (2 wk)	—		—		
	G2	186.32 (68.64)	—	274.58 (137.29) (2 wk)	—		—		
	Cervical flexion-extension (°)								
	G1	47.55 (12.2)	—	59.8 (6.95) (2 wk)	—		—		
	G2	52.2 (7.5)	—	50.35 (11.35) (2 wk)	—		—		
	Cervical rotation (°)								
	G1	56.6 (13)	—	66.8 (2.8) (2 wk)	—		—		
	G2	55.4 (11.9)	—	60.8 (2.65) (2 wk)	—		—		
	Cervical lateral flexion (°)								
	G1	32.95 (10.65)	—	43.45 (7.8) (2 wk)	—		—		
	G2	36.8 (10.5)	—	33.55 (9.2) (2 wk)	—		—		

Stieven et al., 2020 [31]	Pain (NPRS, 0–10)								
	G1	6.31 (0.72)	—	2.17 (0.81) (4 wk)	2.98 (0.63) (12 wk)		3.26 (0.74) (24 wk)		
	G2	6.18 (1.07)	—	3.37 (1.22) (4 wk)	3.52 (0.95) (12 wk)		3.60 (0.56) (24 wk)		
	Disability (NDI, %)								
	G1	26.52 (9.72)	—	22.94 (8.891) (4 wk)	23.08 (11.1) (12 wk)		24.99 (9.04) (24 wk)		
	G2	27.13 (6.42)	—	20.94 (10.4) (4 wk)	23.66 (8.91) (12 wk)		22.86 (7.28) (24 wk)		
	Pain catastrophizing (PCS)								
	G1	23.67 (9.51)	—	22.17 (6.04) (4 wk)	19.07 (7.89) (12 wk)		19.18 (6.47) (24 wk)		
	G2	20.97 (8.56)	—	21.08 (8.83) (4 wk)	21.26 (9.41) (12 wk)		18.94 (8.06) (24 wk)		

Gallego-Sendarrubias et al., 2020 [28]	Pain (VAS, 0–10)						
	G1	6.66 (1.4)	3.19 (1.6)	1.77 (1.4) (4 wk)	—		—
	G2	6.17 (1.6)	4.15 (1.9)	3.34 (1.2) (4 wk)	—		—
	PPT (kPa)						
	G1	171.61 (39.22)	303.02 (78.45)	355.97 (98.06) (4 wk)	—		—
	G2	184.36 (49.033)	253.01 (78.45)	219.66 (68.64) (4 wk)	—		—
	Disability (NDI)						
	G1	28.95 (10.2)	76.81 (12.1)	4.61 (6.2) (4 wk)	—		—
	G2	23.69 (9.8)	73.13 (10.5)	11.52 (7.3) (4 wk)	—		—
	Cervical flexion (°)						
	G1	67.89 (13.5)	64.19 (12.0)	78.38 (9.5) (4 wk)	—		—
	G2	69.25 (11.1)	61.92 (11.5)	70.58 (9.4) (4 wk)	—		—
	Cervical extension (°)						
	G1	52.32 (14.6)	82.60 (14.5)	69.09 (11.3) (4 wk)	—		—
	G2	55.68 (11.5)	79.11 (13.2)	62.40 (10.4) (4 wk)	—		—
	Right rotation (°)						
	G1	74.28 (18.1)	81.45 (13.7)	87.55 (8.1) (4 wk)	—		—
	G2	74.60 (15.3)	79.13 (11.5)	78.79 (10.9) (4 wk)	—		—
	Left rotation (°)						
	G1	71.79 (17.7)	82.02 (14.1)	87.19 (8.4) (4 wk)	—		—
	G2	73.81 (11.8)	79.12 (12.35)	78.89 (10.1) (4 wk)	—		—

Gallego-Sendarrubias et al., 2020 [28]	Cervical rotation (°) (mean calculated)						
	G1	73.03 (17.9)	59.60 (11.9)	87.37 (8.25) (4 wk)	—		—
	G2	74.2 (13.55)	56.28 (11.9)	78.84 (10.5) (4 wk)	—		—
	Right cervical lateral flexion (°)						
	G1	47.72 (14.3)	62.51 (10.5)	68.45 (12.5) (4 wk)	—		—
	G2	49.04 (13.1)	59.87 (10.6)	56.25 (13.0) (4 wk)	—		—
	Left cervical lateral flexion (°)						
	G1	53.64 (10.8)	61.05 (11.2)	70.11 (10.8) (4 wk)	—		—
	G2	55.43 (11.0)	58.07 (11.25)	59.77 (9.9) (4 wk)	—		—
	Cervical lateral flexion (mean calculated)						
	G1	50.68 (12.55)	—	69.28 (11.65) (4 wk)	—		—
	G2	52.23 (12.05)	—	58.01 (11.45) (4 wk)	—		—
*Dry needling combined with other interventions vs. dry needling alone*								

León-Hernández et al., 2016 [24]	Pain (VAS, 0–10)+							
	G1	5.00 (4.00–6.00)	2.50 (1.00–4.00)	—	—	—		
	G2	5.00 (3.50–6.00)	2.00 (1.00–5.00)	—	—	—		
	Disability (NDI)+							
	G1	9.50 (8.00–13.00)	6.50 (3.25–10.00)	—	—	—		
	G2	11.00 (7.00–14.50)	6.00 (4.00–14.00)	—	—	—		
	Cervical flexion (°)							
	G1	52.05 (12.24)	53.76 (12.07)	—	—	—		
	G2	52.42 (11.72)	51.07 (12.21)	—	—	—		
	Cervical extension (°)							
	G1	58.62 (11.80)	62.98 (10.90)	—	—	—		
	G2	58.11 (12.44)	60.26 (13.73)	—	—	—		

León-Hernández et al., 2016 [24]	Cervical left lateral flexion (°)							
	G1	38.16 (9.36)	41.80 (9.63)	—	—	—		
	G2	39.05 (8.01)	39.77 (9.18)	—	—	—		
	Cervical right lateral flexion (°)							
	G1	37.55 (9.11)	40.75 (9.91)	—	—	—		
	G2	38.77 (10.46)	40.59 (9.67)	—	—	—		
	Cervical lateral flexion (mean calculated)							
	G1	37.85 (9.23)	41.27 (9.77)	—	—	—		
	G2	38.91 (9.23)	40.18 (9.42)	—	—	—		
	Cervical left rotation (°)							
	G1	59.31 (13.72)	59.59 (11.98)	—	—	—		
	G2	61.40 (12.82)	64.33 (8.58)	—	—	—		
	Cervical right rotation (°)							
	G1	60.50 (10.73)	61.18 (10.9)	—	—	—		
	G2	60.36 (10.24)	62.22 (7.42)	—	—	—		
	Cervical rotation (mean calculated)							
	G1	59.90 (12.22)	60.38 (11.44)	—	—	—		
	G2	60.88 (11.53)	63.27 (8)	—	—	—		

Valiente-Castrillo et al., 2020 [25]	Pain (VAS, 0–10)							
	G1	5.79 (1.89)	—	2.38 (1.85) (4 wk)	3.00 (1.73) (12 wk)	—		
	G2	5.52 (1.80)	—	1.17 (1.12) (4 wk)	2.47 (2.31) (12 wk)	—		
	G3 (N/A)	5.26 (1.46)	—	3.85 (2.38) (4 wk)	3.91 (2.50) (12 wk)	—		
	Disability (NDI)							
	G1	17.45 (4.94)	—	12.00 (5.68) (4 wk)	11.0 (6.06) (12 wk)	—		
	G2	15.80 (4.62)	—	7.19 (6.56) (4 wk)	7.57 (5.33) (12 wk)	—		
	G3 (N/A)	16.78 (5.32)	—	13.21 (7.26) (4 wk)	13.78 (8.78) (12 wk)	—		
	Pain catastrophizing (PCS)							
	G1	16.95 (11.39)	—	12.10 (11.01) (4 wk)	11.75 (10.48) (12 wk)	—		
	G2	16.55 (12.36)	—	7.33 (7.90) (4 wk)	6.61 (7.86) (12 wk)	—		
	G3 (N/A)	19.41 (11.37)	—	12.70 (10.95) (4 wk)	14.70 (10.26) (12 wk)	—		

^+^Data are expressed as median and interquartile range. They were converted to mean and standard deviation in the forest plots.

**Table 4:** Level of evidence (GRADE) for dry needling on pain intensity, pressure pain sensitivity, and cervical range of motion in patients with neck pain.

Number of studies	Risk of bias	Inconsistency	Indirectness of evidence	Imprecision	Publication bias	Quality of evidence	MD or SMD (95% CI)
Effects of the inclusion of dry needling on neck pain intensity							
** **Immediate follow-up (less than 1 week after single session)							
Overall effect (*n* = 2)	No	Serious (*I*^2^ = 54%)	No	Very serious	No	Very low	MD −0.55 (−1.64 to 0.55)
DN plus other therapy vs. others (n = 1)	No	No	No	Serious	No	Low	MD −0.96 (−1.61 to −0.31)^*∗*^
DN plus other therapy vs. DN alone (*n* = 1)	No	No	No	Very serious	No	Low	MD 0.21 (−1.20 to 1.62)

** **Short-term follow-up (1–12 weeks after intervention)							
Overall effect (*n* = 7)	No	Very serious (*I*^2^ = 94%)	No	No	No	Low	MD −1.76 (−2.66 to −0.86)^*∗*^
DN plus other therapy vs. other (*n* = 6)	No	Very serious (*I*^2^ = 95%)	No	No	No	Low	MD −1.84 (−2.83 to −0.85)^*∗*^
DN plus other therapy vs. DN alone (*n* = 1)	No	No	No	Very serious	No	Low	MD −1.21 (−2.15 to −0.27)^*∗*^

** **Midterm follow-up (12–24 weeks after intervention)							
Overall effect (*n* = 3)	No	No (*I*^2^ = 0%)	No	Serious	No	Moderate	MD −0.52 (−0.79 to −0.25)^*∗*^
DN plus other therapy vs. others (*n* = 2)	No	No (*I*^2^ = 0%)	No	Serious	No	Moderate	MD −0.52 (−0.80 to −0.24)^*∗*^
DN plus other therapy vs. DN alone (*n* = 1)	No	No	No	Serious	No	Moderate	MD −0.53 (−1.78 to 0.72)

** **Long-term follow-up (more than 24 weeks after intervention)							
Overall effect (*n* = 3)	No	Very serious (*I*^2^ = 98%)	No	No	No	Low	MD −1.11 (−2.56 to 0.35)
DN plus other therapy vs. others (*n* = 3)	No	Very serious (*I*^2^ = 98%)	No	No	No	Low	MD −1.11 (−2.56 to 0.35)

Effects of the inclusion of dry needling on pain-related disability							
** **Short-term follow-up (1–12 weeks after intervention)							
Overall effect (*n* = 6)	No	Very serious (*I*^2^ = 81%)	No	No	No	Low	SMD −0.45 (−0.87 to −0.03)^*∗*^
DN plus other therapy vs. others (*n* = 5)	No	Very serious (*I*^2^ = 84%)	No	No	No	Low	SMD −0.39 (−0.87 to 0.08)
DN plus other therapy vs. DN alone (*n* = 1)	No	No	No	Serious	No	Moderate	SMD −0.77 (−1.40 to −0.13)^*∗*^

** **Midterm follow-up (12–24 weeks after intervention)							
Overall effect (*n* = 3)	No	No (*I*^2^ = 11%)	No	Very serious	No	Low	SMD −0.16 (−0.44 to 0.11)
DN plus other therapy vs. others (*n* = 2)	No	No (*I*^2^ = 0%)	No	Very serious	No	Low	SMD −0.07 (−0.35 to 0.21)
DN plus other therapy vs. DN alone (*n* = 1)	No	No	No	Very serious	No	Low	SMD −0.59 (−1.22 to 0.04)

** **Long-term follow-up (more than 24 weeks after intervention)							
Overall effect (*n* = 3)	No	Very serious (*I*^2^ = 88%)	No	No	No	Low	SMD −0.32 (−0.97 to 0.29)
DN plus other therapy vs. others (*n* = 3)	No	Very serious (*I*^2^ = 88%)	No	No	No	Low	SMD −0.32 (−0.97 to 0.29)

Effects of the inclusion of dry needling on pressure pain thresholds							
** **Immediate follow-up (less than 1 week after single session)							
Overall effect (*n* = 3)	No	Serious (*I*^2^ = 79%)	No	Serious	No	Low	MD 40.26 (−20.42 to 100.94)
DN plus other therapy vs. others (*n* = 1)	No	No	No	Serious	No	Moderate	MD 50.01 (19.20 to 80.82)^*∗*^
DN plus other therapy vs. DN alone (*n* = 2)	No	Very serious (*I*^2^ = 80%)	No	Very serious	No	Very low	MD 69.18 (−107.93 to 246.28)

** **Short-term follow-up (1–12 weeks after intervention)							
Overall effect (*n* = 4)	No	Very serious (*I*^2^ = 91%)	No	No	No	Low	MD 110.43 (26.71 to 194.15)^*∗*^
DN plus other therapy vs. others (*n* = 4)	No	Very serious (*I*^2^ = 91%)	No	No	No	Low	MD 110.43 (26.71 to 194.15)^*∗*^

** **Midterm follow-up (12–24 weeks after intervention)							
Overall effect (*n* = 1)	No	No	No	Very serious	No	Low	MD 32.10 (−21.68 to 85.88)
DN plus other therapy vs. others (*n* = 1)	No	No	No	Very serious	No	Low	MD 32.10 (−21.68 to 85.88)

** **Long-term follow-up (more than 24 weeks after intervention)							
Overall effect (*n* = 2)	No	Very serious (*I*^2^ = 88%)	No	Very serious	No	Very low	MD 50.09 (−64.61 to 164.78)
DN plus other therapy vs. others (*n* = 2)	No	Very serious (*I*^2^ = 88%)	No	Very serious	No	Very low	MD 50.09 (−64.61 to 164.78)

Effects of the inclusion of dry needling on cervical flexion range of motion							
** **Immediate follow-up (less than 1 week after single session)							
Overall effect (*n* = 2)	No	No (*I*^2^ = 0%)	No	Very serious	No	Low	MD 3.34 (−0.28 to 6.97)
DN plus other therapy vs. others (*n* = 1)	No	No	No	Very serious	No	Low	MD 3.68 (−0.79 to 8.15)
DN plus other therapy vs. DN alone (*n* = 1)	No	No	No	Very serious	No	Low	MD 2.69 (−3.51 to 8.89)

** **Short-term follow-up (1–12 weeks after intervention							
Overall effect (*n* = 4)	No	Serious (*I*^2^ = 55%)	No	No	No	Moderate	MD 6.01 (2.86 to 9.16)^*∗*^
DN plus other therapy vs. others (*n* = 4)	No	Serious (*I*^2^ = 55%)	No	No	No	Moderate	MD 6.01 (2.86 to 9.16)^*∗*^

** **Long-term follow-up (more than 24 weeks after intervention)							
Overall effect (*n* = 2)	No	Serious (*I*^2^ = 74%)	No	Serious	No	Low	MD 2.89 (−4.67 to 10.45)
DN plus other therapy vs. others (*n* = 2)	No	Serious (*I*^2^ = 74%)	No	Serious	No	Low	MD 2.89 (−4.67 to 10.45)

Effects of the inclusion of dry needling on cervical extension range of motion							
** **Immediate follow-up (less than 1 week after single session)							
Overall effect (*n* = 2)	No	No (*I*^2^ = 0%)	No	Very serious	No	Low	MD 2.43 (−1.30 to 6.16)
DN plus other therapy vs. others (*n* = 1)	No	No	No	Very serious	No	Low	MD 2.27 (−2.35 to 6.89)
DN plus other therapy vs. DN alone (*n* = 1)	No	No	No	Very serious	No	Low	MD 2.72 (−3.59 to 9.03)

** **Short-term follow-up (1–12 weeks after intervention)							
Overall effect (*n* = 4)	No	Serious (*I*^2^ = 59%)	No	No	No	Moderate	MD 5.36 (2.00 to 8.72)^*∗*^
DN plus other therapy vs. others (*n* = 4)	No	Serious (*I*^2^ = 59%)	No	No	No	Moderate	MD 5.36 (2.00 to 8.72)^*∗*^

Long-term follow-up (more than 24 weeks after intervention)							
Overall effect (*n* = 2)	No	Very serious (*I*^2^ = 85%)	No	Serious	No	Very low	MD 1.67 (−7.94 to 11.27)
DN plus other therapy vs. others (*n* = 2)	No	Very serious (*I*^2^ = 85%)	No	Serious	No	Very low	MD 1.67 (−7.94 to 11.27)

Effects of the inclusion of dry needling on cervical rotation range of motion							
** **Immediate follow-up (less than 1 week after single session)							
Overall effect (*n* = 2)	No	Serious (*I*^2^ = 59%)	No	Very serious	No	Very low	MD −0.03 (−5.71 to 5.64)
DN plus other therapy vs. others (*n* = 1)	No	No	No	Very serious	No	Low	MD 2.90 (−2.33 to 8.13)
DN plus other therapy vs. DN alone (*n* = 1)	No	No	No	Very serious	No	Low	MD −2.89 (−7.94 to 2.16)

** **Short-term follow-up (1–12 weeks after intervention)							
Overall effect (*n* = 4)	No	No (*I*^2^ = 32%)	No	No	No	High	MD 6.34 (4.66 to 8.03)^*∗*^
DN plus other therapy vs. others (*n* = 4)	No	No (*I*^2^ = 32%)	No	No	No	High	MD 6.34 (4.66 to 8.03)^*∗*^

** **Long-term follow-up (more than 24 weeks after intervention)							
Overall effect (*n* = 2)	No	Serious (*I*^2^ = 74%)	No	Serious	No	Low	MD 4.25 (−3.78 to 12.28)
DN plus other therapy vs. others (*n* = 2)	No	Serious (*I*^2^ = 74%)	No	Serious	No	Low	MD 4.25 (−3.78 to 12.28)

Effects of the inclusion of dry needling on cervical lateral flexion range of motion							
** **Immediate follow-up (less than 1 week after single session)							
Overall effect (*n* = 2)	No	No (*I*^2^ = 0%)	No	Very serious	No	Low	MD 2.13 (−1.14 to 5.41)
DN plus other therapy vs. others (*n* = 1)	No	No	No	Very serious	No	Low	MD 2.98 (−1.43 to 7.39)
DN plus other therapy vs. DN alone (*n* = 1)	No	No	No	Very serious	No	Low	MD 1.09 (−1.14 to 5.41)

Short-term follow-up (1–12 weeks after intervention)							
Overall effect (*n* = 3)	No	Serious (*I*^2^ = 63%)	No	Serious	No	Low	MD 8.55 (5.01 to 12.10)^*∗*^
DN plus other therapy vs. others (*n* = 3)	No	Serious (*I*^2^ = 63%)	No	Serious	No	Low	MD 8.55 (5.01 to 12.10)^*∗*^

** **Long-term follow-up (more than 24 weeks after intervention)							
Overall effect (*n* = 1)	No	No	No	Very serious	No	Low	MD 5.89 (3.72 to 8.06)^*∗*^
DN plus other therapy vs. others (*n* = 1)	No	No	No	Very serious	No	Low	MD 5.89 (3.72 to 8.06)^*∗*^

Effects of the inclusion of dry needling on pain catastrophizing							
** **Short-term follow-up (1–12 weeks after intervention)							
Overall effect (*n* = 3)	No	Serious (*I*^2^ = 76%)	No	Very serious	No	Very low	MD −3.01 (−8.33 to 2.30)
DN plus other therapy vs. others (*n* = 2)	No	Very serious (*I*^2^ = 85%)	No	Very serious	No	Very low	MD −2.44 (−9.95 to 5.07)
DN plus other therapy vs. DN alone (*n* = 1)	No	No	No	Very serious	No	Low	MD −4.77 (−10.66 to 1.12)

** **Midterm follow-up (12–24 weeks after intervention)							
Overall effect (*n* = 3)	No	No (*I*^2^ = 0%)	No	Serious	No	Moderate	MD −3.34 (−5.77 to −0.91)^*∗*^
DN plus other therapy vs. others (*n* = 2)	No	No (*I*^2^ = 0%)	No	Serious	No	Moderate	MD −2.94 (5.63 to −0.25)^*∗*^
DN plus other therapy vs. DN alone (*n* = 1)	No	No	No	Very serious	No	Low	MD −5.14 (−10.83 to 0.55)

** **Long-term follow-up (more than 24 weeks after intervention)							
Overall effect (*n* = 2)	No	Serious (*I*^2^ = 65%)	No	Very serious	No	Very low	MD −1.71 (−6.36 to 2.94)
DN plus other therapy vs. others (*n* = 2)	No	Serious (*I*^2^ = 65%)	No	Very serious	No	Very low	MD −1.71 (−6.36 to 2.94)

^*∗*^Statistically significant (*P* < 0.05). Risk of bias: No, most information is from results at low risk of bias; Serious, crucial limitation for one criterion or some limitations for multiple criteria, sufficient to lower confidence in the estimate of the effect; Very serious, crucial limitation for one or more criteria sufficient to substantially lower confidence in the estimate of the effect. Inconsistency: Serious, *I*^2^> 40%; Very serious, *I*^2^>80%. Indirectness of evidence, no indirectness of evidence was found in any study. Imprecision (based on sample size): Serious, *n* < 250 subjects; Very serious, *n* < 250, and the estimated effect is little or absent. Publication bias (based on funnel plots), no publication bias was found. Funnel plots are not shown because of the small number of trials.

## Data Availability

No data are publicly available since this is a systematic review and meta-analysis.

## Supplementary Materials

Supplementary Materials Supplementary Table 1: database formulas during literature search; Supplementary Table 2: characteristics of the dry needling intervention of the included studies; Supplementary Table 3: adverse events described in the included studies.
