# Supplementary material for: Is Dry Needling Effective When Combined with Other Therapies for Myofascial Trigger Points Associated with Neck Pain Symptoms? A Systematic Review and Meta-Analysis
Source: Pain Res Manag. 2021 Feb 2;2021:8836427. doi: 10.1155/2021/8836427 (PMC7872772; doi:10.1155/2021/8836427)
Supplement: Supplementary Materials — Supplementary Table 1: database formulas during literature search; Supplementary Table 2: characteristics of the dry needling intervention of the included studies; Supplementary Table 3: adverse events described in the included studies. [file 8836427.f1.zip › 8836427.f1/Suplementary Table 1.docx]

**Supplementary Table 1:** Database formulas during literature search

| **PubMed Search Formula**  #1 "Dry Needling" [Mesh] OR "Trigger Point Acupuncture" [Title/Abstract] OR "Needling Therapy" [Title/Abstract] OR "Intramuscular Stimulation" [Title/Abstract]  #2 ““Control Groups” [Mesh] OR “Physical Therapy Modalities” [Mesh]  #3 "Neck Pain" [Mesh] OR "Non-Specific Neck Pain" [Title/Abstract] OR "Cervicalgia" [Title/Abstract] OR "Cervical Pain" [Title/Abstract] OR "Mechanical Neck Pain" [Title/Abstract] OR “Myofascial Neck Pain” [Title/Abstract]  #4 #1 AND #2 AND #3 |
| --- |
| **CINAHL / Medline (via EBSCO) Search Formula**  #1 "Dry Needling" OR "Trigger Point Acupuncture" OR "Needling Therapy" OR "Intramuscular Stimulation"  #2 “Control Groups” OR “Physical Therapy Modalities”  #3 "Neck Pain" OR "Non-Specific Neck Pain" OR "Cervicalgia" OR "Cervical Pain" OR "Mechanical Neck Pain" OR “Myofascial Neck Pain”  #4 #1 AND #2 AND #3 |
| **SCOPUS Search Formula**  TITLE-ABS-KEY (Dry Needling" OR "Trigger Point Acupuncture" OR "Needling Therapy" OR "Intramuscular Stimulation") AND TITLE-ABS-KEY (“Control Groups” OR “Physical Therapy Modalities”) AND TITLE-ABS-KEY (“Neck Pain" OR "Non-Specific Neck Pain" OR "Cervicalgia" OR "Cervical Pain" OR "Mechanical Neck Pain" OR “Myofascial Neck Pain”) |
| **PEDro Search Formula**  Abstract & Title: Neck Pain, Myofascial Pain Syndrome  Therapy: Dry Needling  Method: Clinical trial  When Searching: AND |
| **WOS Search Formula**  ("Dry Needling" OR "Trigger Point Acupuncture" OR "Needling Therapy" OR "Intramuscular Stimulation") AND (“Control Groups” OR “Physical Therapy Modalities”) AND (“Neck Pain" OR "Non-Specific Neck Pain" OR "Cervicalgia" OR "Cervical Pain" OR "Mechanical Neck Pain" OR “Myofascial Neck Pain”) |
| **Cochrane Library Search Formula**  #1 Mesh: Dry Needling  #2 Mesh: Physical Therapy Modalities  #3 Mesh: Neck Pain  #4 Trigger Point Acupuncture  #5 Needling Therapy  #6 Intramuscular Stimulation  #7 Mesh: Control Groups  #8 Non-Specific Neck Pain  #9 Cervicalgia  #10 Cervical Pain  #11 Mechanical Neck Pain  #12 Myofascial Neck Pain  #13 #1 OR #4 OR #5 OR #6  #14 #2 OR #7  #15 #3 OR #8 OR #9 OR #10 OR #11 OR #12  #16 #13 AND #14 AND #15 |
