# Supplementary material for: Is Dry Needling Effective When Combined with Other Therapies for Myofascial Trigger Points Associated with Neck Pain Symptoms? A Systematic Review and Meta-Analysis
Source: Pain Res Manag. 2021 Feb 2;2021:8836427. doi: 10.1155/2021/8836427 (PMC7872772; doi:10.1155/2021/8836427)
Supplement: Supplementary Materials — Supplementary Table 1: database formulas during literature search; Supplementary Table 2: characteristics of the dry needling intervention of the included studies; Supplementary Table 3: adverse events described in the included studies. [file 8836427.f1.zip › 8836427.f1/Suplementary Table 2.docx]

| **Supplementary Table 2:** Characteristics of the dry needling intervention of the included studies | | | | | | | | | | | | |
| --- | --- | --- | --- | --- | --- | --- | --- | --- | --- | --- | --- | --- |
| **Study** | **Group** | **TrP criteria** | **Technique used** | **No. punctures for patient in intervention** | **Needle approach (targeted muscles or tendon)** | **Gauge (mm)** | **Depth (mm)** | **Time of DN** | **Frequency of incisions (Hz)** | **Number of incisions in every needle intervention** | **LTR** | **Therapist that performed intervention** |
| Tough et al. 2010 | G1: Acupuncture  G2: Sham-Acupuncture | Yes  Yes | ‘sparrow pecking’ technique  ‘sparrow pecking’ technique | NR  NR | All muscle with TrP with pain  All muscle with TrP with pain | 0.25x30-40 mm  0.25x30-40 mm | NR  NR | NR  NR | NR  NR | 5-6 times  5-6 times | No  No | Physical Therapist  Physical Therapist |
| Sterling et al. 2015 | G1: DN  G2: Sham-DN | No  No | “pecking” or  “twirling” movements of the needle  “pecking” or  “twirling” movements of the needle | At most 5  At most 5 | Trapezius, levator scapulae, splenius capitis, semispinalis, and spinalis capitis muscles  Trapezius, levator scapulae, splenius capitis, semispinalis, and spinalis capitis muscles | NR  NR | NR  NR | 30 min  30 min | NR  NR | Each muscle could undergo 2 to 3 needle insertions  Each muscle could undergo 2 to 3 needle insertions | No  No | Physical Therapist  Physical Therapist |
| Cerezo-Tellez et al. 2016 | G1: DN | Yes | Hong | 1 | Trapezius (all three divisions), cervical multifidi, splenius cervicis, and levator scapulae muscles | 0.32x40 mm | Into of active TrP | NR | NR | Four to five local twitch responses | Yes | Physical Therapist |
| Cerezo-Tellez et al. b 2016 | G1: DN | Yes | Hong | 1 | Trapezius (all three divisions) | 0.32x40 mm | Into of active TrP | NR | NR | Four local twitch responses | Yes | Physical Therapist |
| León-Hernández et al. 2016 | G1: DN  G2: DN | Yes  Yes | Hong  Hong | 1  1 | Upper trapezius  Upper trapezius | 0.32x40 mm  0.32x40 mm | Inside of muscle  Inside of muscle | NR  NR | NR  NR | Until two local twitch responses were elicited  until two local twitch responses were elicited | Yes  Yes | Physical Therapist  Physical Therapist |
| Gallego-Sendarrubias et al. 2020 | G1: DN  G2: Sham-DN | Yes  Yes | Hong  Hong with blunt needle | 3  3 | Upper trapezius and levator scapulae  Upper trapezius and levator scapulae | 0.32x40 mm  0.32x40 mm | Until muscle  Until muscle | 10 sg  10 sg | NR  NR | Until a local  twitch response was obtained  Until a local  twitch response was obtained | Yes  No | Physical Therapist  Physical Therapist |
| Stieven et al. 2020 | G1: DN | Yes | Hong | 6 | Upper and middle trapezius, cervical multifidi, splenius cervicis, and levator scapulae muscles | 0.25x40 mm | 10-15 mm | 30 -60 sg | NR | Until to obtain up to six additional twitch responses | Yes | Physical Therapist |
| Valiente-Castrillo et al. 2020 | G1: DN | Yes | Hong | 4 | Upper trapezius, levator scapulae,  cervical multifidus, and cervical splenius | 032x40 mm | Until muscle | NR | NR | Until to obtain five local twitch responses | Yes | Physical Therapist |

DN: Dry Needling; G: Group; LTR: Local twitch response;
