# Supplementary material for: Is Dry Needling Effective When Combined with Other Therapies for Myofascial Trigger Points Associated with Neck Pain Symptoms? A Systematic Review and Meta-Analysis
Source: Pain Res Manag. 2021 Feb 2;2021:8836427. doi: 10.1155/2021/8836427 (PMC7872772; doi:10.1155/2021/8836427)
Supplement: Supplementary Materials — Supplementary Table 1: database formulas during literature search; Supplementary Table 2: characteristics of the dry needling intervention of the included studies; Supplementary Table 3: adverse events described in the included studies. [file 8836427.f1.zip › 8836427.f1/Suplementary Table 3.docx]

**Supplementary Table 3:** Adverse events described in the included studies

| Tough et al. 2010 | A temporary increase in pain (lasting no longer than the day of treatment) was reported by 16/20 (80%) patients who received the real needling and by 9/20 (43%) patients who received the sham procedure. These symptoms resolved spontaneously within 24-48 hours without further treatment. |
| --- | --- |
| Sterling et al. 2015 | Two patients within the dry needling and exercise group and two within the sham-needling and exercise group experienced an exacerbation of neck pain symptoms. None of the patients withdrew from the trial because adverse effects. These symptoms spontaneously within 24-48 hours without further treatment. |
| León-Hernández et al. 2016 | Most patients experienced post-needling soreness. The application of Percutaneous Electrical Nerve Stimulation (PENS) reduced the duration of post-needling soreness. |
| Cerezo-Tellez et al. 2016 | Soreness and local hemorrhages occurred after dry needling in some cases, but they resolved within one week without further treatment. |
| Cerezo-Tellez et al. 2016 (b) | No data about adverse events were provided |
| Gallego-Sendarrubias et al. 2020 | No adverse effects occurred during the study. |
| Stieven et al. 2020 | Six patients (10.5%) in the physical therapy and 8 patients (13.8%) in the dry needling plus physical therapy experienced mild adverse events. Two patients experienced temporary posterior neck pain exacerbation, five neck-shoulder pain exacerbation and one temporary headache in the dry needling group |
| Valiente-Castrillo et al. 2020 | A total of 90% of patients presented post-needling soreness during the follow-up period. These symptoms resolved spontaneously within 24-48 hours without further treatment. No  other adverse effects were reported. |
